# Overwork-Induced Sudden Death: A Sword of Damocles Hanging over Chinese Medical Staff

**Published:** 2020-02

**Authors:** Ce YANG, Zhilan CHEN, Liyong CHEN

**Affiliations:** 1. State Key Laboratory of Trauma, Burn and Combined Injury, Research Institute of Surgery, Daping Hospital, Third Military Medical University, Chongqing, China; 2. Reproductive Medical Center, Wuhan Central Hospital, Wuhan, China; 3. Department of Anesthesiology, Research Institute of Surgery and Daping Hospital, Third Military Medical University, Chongqing, China

## Dear Editor-in-Chief

We carried out a selective cross-section research of sudden death (SD) in Chinese medical staff over the past 10 years.

**Fig. 1:**
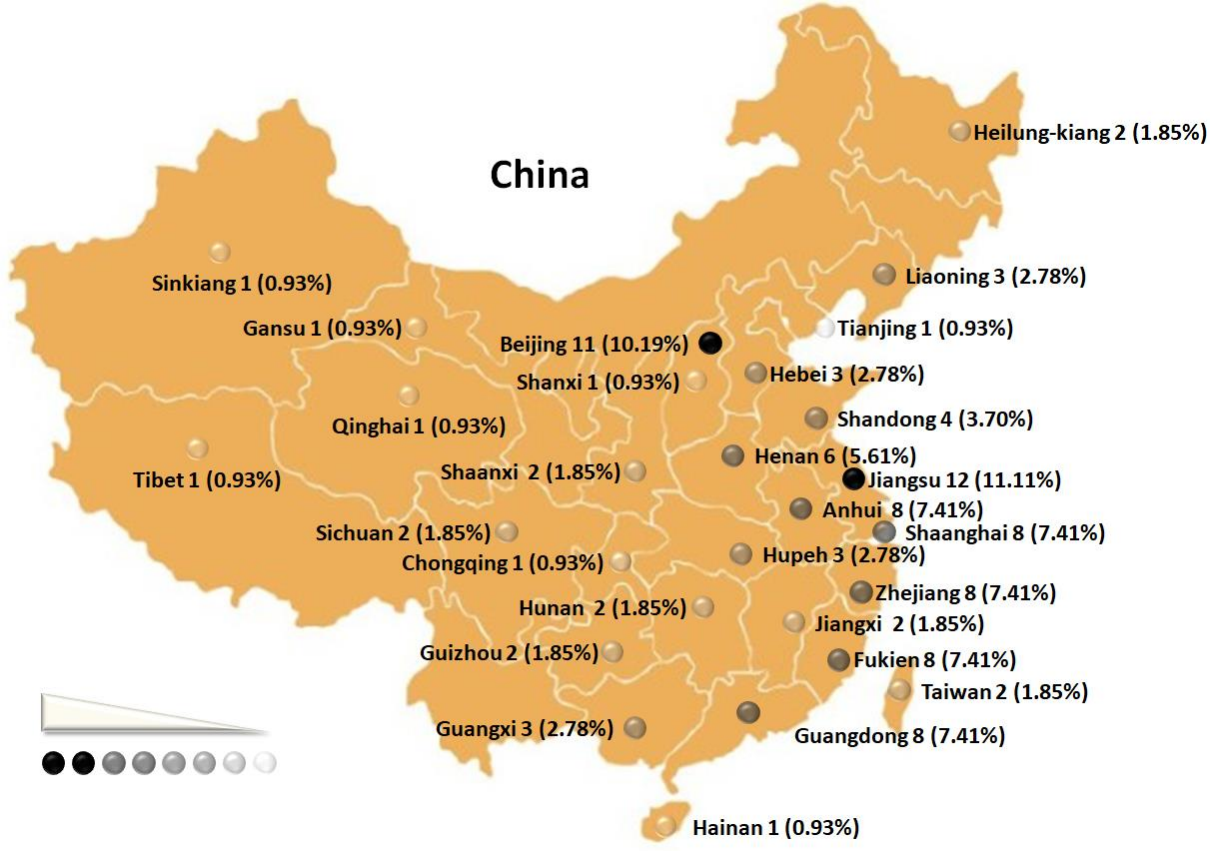
Regional distribution of sudden death among Chinese medical staff from 2008 to 2018

The subjects of this research between Jan 1, 2008 and Apr 30, 2018 were the medical staff remaining in positions. Field research took place in all the administrative territories in China (Fig. 1).

Growing evidence in the past 10 years witnessed the escalation of sudden deaths in Chinese medical staff. Between Jan 1, 2008 and Apr 30, 2018, 107 sudden death accidents with a 2.80% rate of rescue occurred were reported for Chinese medical staff under the stressful overwork, maybe a tip of a larger iceberg for their health status. It happened among young Chinese physicians aged (39.67±9.46 yr; male: female =7.75: 1). It struck surgeons (39.25%), anesthetists (19.63%) or internists (16.82%) most in developed regions in China. The confirmed death causes were cardiac arrhythmia, dissecting aneurysm of artery and rupture of cerebral blood vessels ascribed to stress. The reasons for an upward trend of sudden deaths in Chinese physicians are complicated. First, China occupies only 2% of world’s medical resources while possessing 22% of global total population (1), aggravated by a flawed hierarchical medical system. The boosted appeal for Chinese public forced the physicians overworking. Regarding the data from Chinese white paper of physician practice condition in 2017, the average hospital duty hours reached 50.9 and 49.79 per week for male and female physicians far beyond the national legal working hours of 40.00 a week. Nearly a quarter of Chinese physicians enjoyed the statutory annual leave, far less than those in developed countries.

Second, the promotion schema for Chinese physicians sets a seemingly reasonable criterion of National Science Foundation and articles published in Science Citation Index journals (2). Such biased evaluation induced Manuscript the burn-out working pattern of 5 plus 2 d and day plus night per work. Long working hours and excessive work stress constitute an adelomorphic killer. Third, deterioration of patient-doctor relationships from brainsick patients and their relatives resulted in persisting intellectual state and high tension in an imbalance of neuroendocrine immune network (3). The exhaustion from body and soul finally came into being.

Thus, the quick development of primary medical care and medical resource redistribution especially under the medical alliance is urgent. Academic assessment and social benefits should accommodate clinical competence essentially. The recruitment of excellent medical graduates after qualification may fill in the huge medical gap with high quality. The compulsive furloughs system is needed legally. Additionally, the rational decoupling of remuneration and medical income may relieve the excessive workload. Regarding the updated Physician’s Pledge (4), the clause “I will attend to my own health” is to provide high-quality medical services for better public health literacy.
